# Prevalence of wearing-off and dyskinesia among the patients with Parkinson’s disease on levodopa therapy: a multi-center registry survey in mainland China

**DOI:** 10.1186/2047-9158-3-26

**Published:** 2014-12-05

**Authors:** Wei Chen, Qin Xiao, Ming Shao, Tao Feng, Wei-Guo Liu, Xiao-Guang Luo, Xiao-Chun Chen, An-Mu Xie, Chun-Feng Liu, Zhen-Guo Liu, Yi-Ming Liu, Jian Wang, Sheng-Di Chen

**Affiliations:** Department of Neurology, Rui Jin Hospital affiliated to Shanghai Jiao Tong University School of Medicine, Rui Jin 2nd Road 197, Shanghai, 200025 China; Department of Neurology, The First Affiliated Hospital of Guangzhou Medical College, Guangzhou, China; Department of Neurology, Beijing Tiantan Hospital affiliated to Capital Medical University, Beijing, China; Department of Neurology, Nanjing Brain Hospital affiliated to Nanjing Medical University, Nanjing, China; Department of Neurology, The First Hospital of China Medical University, Shenyang, China; Department of Neurology, Union XieHe Hospital, Fujian Medical University, Fuzhou, China; Department of Neurology, The Affiliated Hospital of Medical College Qingdao University, Qingdao, China; Department of Neurology, The Second Affiliated Hospital of Soochow University, Suzhou, China; Department of Neurology, Xinhua Hospital affiliated to Shanghai Jiao Tong University School of Medicine, Shanghai, China; Department of Neurology, Qilu Hospital of Shandong University, Jinan, China; Department of Neurology, Huashan Hospital affiliated to Fudan University, Shanghai, China

**Keywords:** Parkinson’s disease, Wearing-off, Dyskinesia, Epidemiology

## Abstract

**Objective:**

Chronic levodopa (L-dopa) treatment in Parkinson’s disease (PD) is often associated with the development of motor complications, but the corresponding epidemiological data is rare in Chinese PD patients. The present survey was to investigate the prevalence rate of wearing-off (WO) and dyskinesia among the patients with PD in China.

**Methods:**

From May 2012 to October 2012, a 3-step registry survey for wearing off (WO) and dyskinesia patients with PD receiving levodopa therapy was performed simultaneously at 28 movement disorders clinics in China.

**Results:**

There were 1,558 PD patients fulfilling the inclusion criteria. Among them, 1,051 had at least one positive response of 9-item wearing off questionnaire (WOQ-9), 724 and 160 patients were finally diagnosed with WO and dyskinesia by movement disorders specialists, respectively. The overall prevalence rates of WO and dyskinesia were 46.5% (95% CI 44.0% - 48.9%) and 10.3% (95% CI 8.8% - 11.8%), respectively. The mean score of WOQ-9 for those with WO was 3.8 (SD = 1.8), with movement slowness being the most common motor symptoms and pain/aching being the most common non-motor symptoms. Better improvement of motor symptoms (n = 354, 87.8%) and long-term disease control and drug selection (*n* = 288, 71.5%) were the two most frequently considered factors when movement disorders specialists adjusted therapeutic strategies for patients with WO.

**Conclusions:**

This survey provided the first multi-center epidemiological data of motor complications among PD patients on L-dopa therapy from mainland China. WO prevalence rate among Chinese PD patients was in line with, while dyskinesia prevalence rate was lower than previous reports from other Countries.

**Electronic supplementary material:**

The online version of this article (doi:10.1186/2047-9158-3-26) contains supplementary material, which is available to authorized users.

## Introduction

Up to now, Levodopa (L-dopa) is still recognized as the most widely used and effective medication for Parkinson’s disease (PD), but long period therapy is often associated with the development of motor complications such as wearing-off (WO) and dyskinesia, which brought not only challenges for neurologists, but also impaired daily living and poor life quality of PD patients. Generally, the first motor complication is predictable WO in advancing PD, that is, a recurrence of motor and non-motor symptoms preceding scheduled doses of anti-PD medication that usually improved after the next dosage [1]. Early identification of such condition is of great importance for the timely optimized treatment of PD.

Cumulative evidence indicated that approximately 40% patients treated with L-dopa for 4–6 years experienced motor complications in Western Countries [2, 3], whereas, the corresponding epidemiological data was rare in Chinese PD patients. Heterogeneity exists in previous reports from Hong Kong [4] and Suzhou [5], China. Moreover, both of them were based on single center data with limited sample size. Thus, the true nature of motor complications, especially WO in Chinese PD patients deserves further investigation. Since the 9-item wearing-off questionnaire (WOQ-9) is a simple and highly-sensitive diagnostic screening scale for WO [6, 7], we considered that WO was rarely present in patients with zero point on WOQ-9 as recommended by Movement Disorders Society (MDS) in 2011 [8].

Once recognized, WO can be effectively managed via a number of therapeutic options, including adjustments of L-dopa dosage and dosage form, adjunctive therapy with catechol-O-methyltransferase (COMT) inhibitors, monoamine oxidase-B (MAO-B) inhibitors and dopamine agonists. Nevertheless, clinical experience and treatment concepts from movement disorders specialists also play critical roles in the optimized therapy of WO [9, 10]. Since PD is still an incurable disease, current therapy could only improve the symptoms, the long-term outcome should be considered when selecting treatment strategy.

Therefore, we conducted a multi-center registry survey in mainland China to explore the prevalence and characteristics of WO among outpatients with PD receiving L-dopa therapy. In addition, the factors considered by movement disorders specialists when making therapeutic choices for WO patients and the prevalence rate of dyskinesia were also investigated.

## Subjects and methods

### Survey setting

From May, 2012 to October, 2012, this cross-sectional, non-interventional registry study was performed simultaneously at 28 movement disorders clinics in tertiary hospitals located in capital cities with over 1,000 beds and the highest medical and academic levels in China. Patients with PD who were willing to participate, provided written informed consent, and met the entry criteria were enrolled at each clinic. During each visit, firstly, preliminary screening for eligible PD patients was done by study nurses before evaluated by the neurologists; Secondly, WOQ-9 and basic information registry form were completed by the informed patients, it could be completed with the aid of study nurse; Finally, comprehensive case report form (CRF) was recorded for patients with at least one positive item (WOQ-9 ≥ 1). The final diagnoses of WO and dyskinesia were made by movement disorders specialists. WO was accepted as the shortening of the duration of L-dopa benefit to less than 4 h for every L-dopa dose given as reference [11]. Dyskinesia was defined as an involuntary choreatic movement involving the muscles of limbs, neck, trunk, or rarely face during the "on" period of patients who displayed prominent improvement of their parkinsonian symptoms [11].

The study was sponsored by Chinese Parkinson’s Disease & Movement Disorders Society, Neurology Branch of Chinese Medical Association, with approval from the Research Ethics Committee, Rui Jin Hospital affiliated to Shanghai Jiao Tong University School of Medicine, Shanghai, China.

### Survey design

Subjects with clinical diagnosed PD based on the United Kingdom PD Brain Bank Criteria [12], were eligible for inclusion into the study if they had L-dopa medication at least 30 days and written informed consent. Patients with secondary parkinsonism and atypical parkinsonism syndrome (such as multiple system atrophy, progressive supranuclear palsy, dementia of lewy body, cortical basal ganglia degeneration, etc.) were excluded. Subjects were also excluded if they withdraw the survey underway.

### 9-item wearing-off questionnaire

For those eligible PD patients, WOQ-9 was completed, which covered both motor and non-motor symptoms of WO [13]. For each item, patients reported whether a symptom was present and whether it improved after the subsequent dose of anti-PD medications. If both were positive, one score was obtained. WOQ-9 defined the possible presence of WO as at least one score obtained.

### Basic information registry form

For enrolled PD patients, demographic parameters such as age, gender, smoking, education, height, body weight and concomitant diseases were recorded on basic information registry form. Clinical characteristics collected included onset age, disease duration, L-dopa duration and medications history. L-dopa dosage and L-dopa equivalent dosage (LED) were calculated as reported by a previous report [14].

### Case report form (CRF)

CRF was completed for PD patients with more than one positive item of WOQ-9. Besides information collected in basic registry form, the motor subscale of the Unified Parkinson’s Disease Rating Scale (UPDRS-III) was used to evaluate motor function [15]. For patients with motor fluctuation, they were assessed during the "on" stage. Modified Abnormal Involuntary Movement Scale (mAIMS) was used to screen dyskinesia [16]. An Activities of Daily Living (ADL) questionnaire was completed by the patients or their caregivers or spouse, which included six physical and eight instrumental ADLs [17]. If movement disorders specialists considered the current treatment protocol of patients with WO needed adjustment, the factors concerned should be selected below: 1) long-term disease control and drug selection, 2) limited economic condition of patients, 3) better improvement of motor symptoms, 4) prior consideration of tolerance and safety of medications, 5) whether the drug was available in hospitals or not, 6) the price of medications, 7) the patients’ choices, 8) others. All the assessments were performed by movement disorders specialists during face-to-face interviews with the patients. Researchers were systematically trained before the investigation.

### Statistical analysis

Statistical analysis was performed with SPSS, the overall and L-dopa duration specific prevalence rates of WO and dyskinesia were estimated and the corresponding 95% confidence interval (CI) were calculated based on the Poisson distribution. The independent sample *t* test and Chi-square analysis were employed for comparing group means and categorical data, respectively. Pearson correlation analysis was used for evaluating bivariate correlation. All *p* values were 2-sided and values < 0.05 were considered statistically significant.

## Results

### WO prevalence, characteristics and associated factors

**Figure 1:**
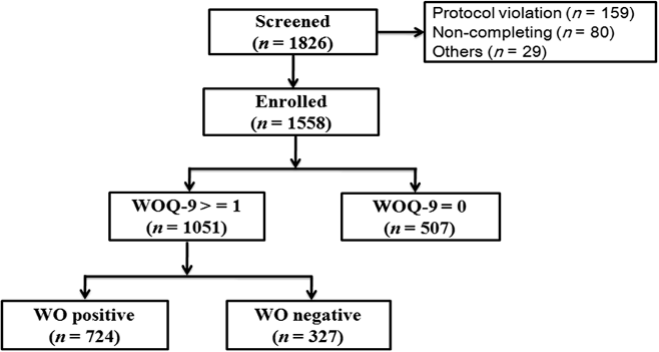
**Flow chart of patients with WO through the survey.** WOQ-9, the 9-item wearing off questionnaire; WO, wearing-off.

Figure 1 described the screening and enrollment protocol through the survey. Of the 1,826 patients screened, 1,558 were enrolled in this study for further evaluation. A total of 268 were excluded, the majority of them were due to protocol violations (*n* = 159) and withdraw underway (*n* = 80). The protocol violation was caused by misdiagnosis of PD. Among these enrolled patients, a total of 507 had negative finding of WOQ-9. Thus, 1,051 with score > =1 of WOQ-9 went into the evaluation stage of movement disorders specialists. The demographic and clinical data of enrolled patients and those with CRF evaluation were summarized in Additional file 1: Table S1. Finally, 724 patients were diagnosed with WO. The overall prevalence rate of WO was 46.5% (95% CI: 44.0% - 48.9%).

**Figure 2:**
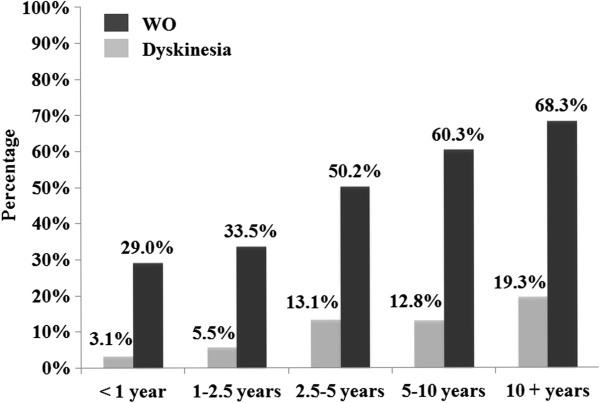
**Percentage of PD with WO and dyskinesia stratified by L-dopa therapy duration (n = 1488).**

The prevalence of WO increased with the prolonged duration of L-dopa treatment (*p* = 0.0013), as shown in Figure 2. 29.0% PD patents with L-dopa duration less than one year experienced WO, the corresponding values were 33.5%, 50.2%, 60.3% and 68.3% for those with L-dopa duration 1–2.5 years, 2.5 - 5 years, 5–10 years and more than 10 years, respectively. The probability of detecting WO by movement disorders specialists was strongly associated with the positive response number of WOQ-9 (*p* = 0.0002).

**Figure 3:**
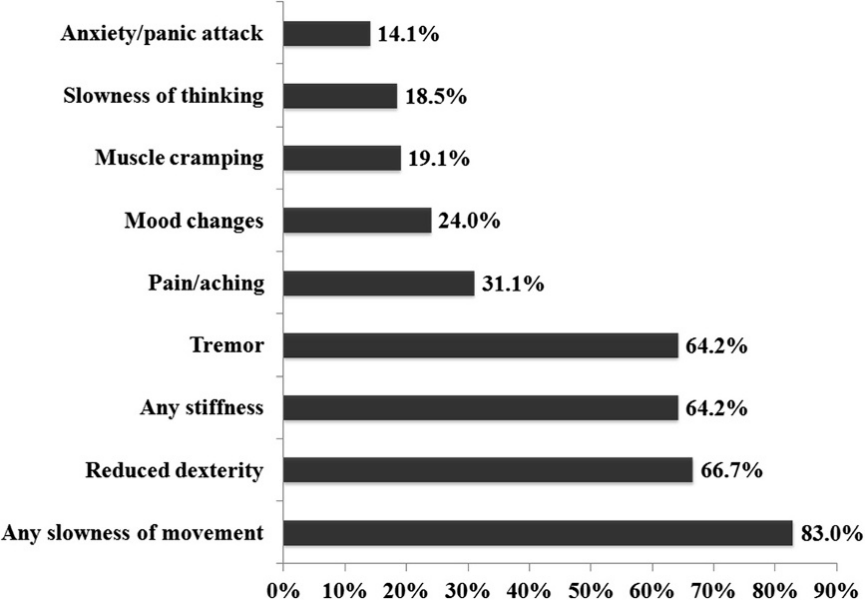
**Frequency of motor and non-motor WO symptoms with respect to WOQ-9 among WO patients (** ***n*** **= 724).**

Among these 724 confirmed patients with WO, the mean score of WOQ-9 was 3.8 (SD = 1.8). Overall, motor WO symptoms outweighed the non-motor WO symptoms (Figure 3). As the most common motor symptoms WO, slowness of movement was identified by up to 83.0% of patients. Whereas, the most common non-motor WO symptoms was pain/aching occupying 31.1% of subjects.

Compared with those without WO, patients with WO had more proportion of female patients (*p* < 0.0422), younger age (*p* < 0.0001), lower weight (*p* < 0.0090), earlier onset age (*p* < 0.0001), longer disease duration (*p* < 0.0001), longer L-dopa therapy duration (*p* < 0.0001), larger L-dopa dosage and LED (*p* < 0.0001), more severe UPDRS-III (*p* < 0.0001) and disabled ADL (*p* < 0.0001). Subgroup analysis for patients with CRF evaluation showed that 19.5% of patients with WO had dyskinesia as confirmed by movement disorders specialists, the proportion of which was significantly higher than that without WO (6.48%) (*p* < 0.0001). With respect to medications, there were more proportions of patients with WO receiving levodopa-benserazide (*p* < 0.0001), levodopa-carbidopa (*p* = 0.0002), Piribedil (*p* = 0.0013) and entacapone (*p* < 0.0001), relative to the WO negative group (Table 1).

**Table 1:** **Differences between enrolled patients with and without WO and dyskinesia**

Items	WO			Dyskinesia		
	With (n = 724)	Without (n = 834)	***P***value	With (n = 160)	Without (n = 1398)	***P***value
**Age, years**			<.0001			0.0016
Mean ± SD	63.8 ± 9.6	66.5 ± 9.3		63.0 ± 9.5	65.5 ± 9.5	
*n* (missing)	710(14)	815(19)		156(4)	1369(29)	
**Gender, Female**			0.0422			0.1959
*n* (%)	347(47.9)	356(42.8)		80(50.0)	623(44.6)	
*n* (missing)	724(0)	832(2)		160(0)	1396(2)	
**Height, cm**			0.2230			0.2203
Mean ± SD	164.1 ± 7.8	164.6 ± 7.7		163.6 ± 8.3	164.4 ± 7.7	
*n* (missing)	717(7)	826(8)		157(3)	1386(12)	
**Weight, kg**			0.0090			<.0001
Mean ± SD	60.9 ± 11.1	62.3 ± 10.7		58.46 ± 10.12	62.01 ± 10.91	
*n* (missing)	720(4)	826(8)		158(2)	1388(10)	
**Onset age, years**			<.0001			<.0001
Mean ± SD	57.3 ± 9.9	62.3 ± 9.5		54.9 ± 9.9	60.5 ± 9.9	
*n* (missing)	711(13)	783(51)		156(4)	1338(60)	
**Disease duration, years**			<.0001			<.0001
Mean ± SD	6.7 ± 4.4	4.2 ± 3.7		8.2 ± 5.5	5.1 ± 4.0	
*n* (missing)	713(11)	796(38)		155(5)	1354(44)	
UPDRS-III*			<.0001			0.1678
Mean ± SD	19.0 ± 8.4	15.6 ± 6.7		18.8 ± 9.1	17.8 ± 7.9	
*n* (missing)	724(0)	327(0)		160(0)	891(0)	
**L-dopa duration, years**			<.0001			<.0001
Mean ± SD	5.1 ± 3.9	3.3 ± 3.5		5.9 ± 4.7	3.9 ± 3.7	
*n* (missing)	703(21)	785(49)		151(9)	1337(61)	
**L-dopa dosage**			<.0001			<.0001
Mean ± SD	478.5 ± 247.0	355.4 ± 191.5		581.7 ± 311.4	393.8 ± 207.7	
*n* (missing)	724(0)	834(0)		160(0)	1398(0)	
**≤400 mg/d** ***n*** **(%)**	359(37.2)	607(62.8)	<.0001	52(5.4)	914(94.6)	<.0001
**400-600 mg/d** *n* (%)	234(56.5)	180(43.5)		54(13.0)	360(87.0)	
**>600 mg/d** *n* (%)	131(73.6)	47(26.4)		54(30.3)	124(69.7)	
**LED**			<.0001			<.0001
Mean ± SD	558.4 ± 281.7	414.4 ± 216.0		707.8 ± 336.9	455.4 ± 234.9	
*n* (missing)	724(0)	834(0)		160(0)	1398(0)	
ADL*			<.0001			<.0001
Mean ± SD	28.4 ± 12.9	21.0 ± 9.6		31.3 ± 13.6	25.1 ± 12.0	
*n* (missing)	721(3)	326(1)		160(0)	887(4)	
**Medications for PD,** ***n*** **(%)**						
Levodopa-benserazide	647(89.4)	682(81.8)	<.0001	136(85.0)	1193(85.3)	0.9094
Levodopa-carbidopa	285(39.4)	253(30.3)	0.0002	87(54.4)	451(32.3)	<.0001
Pramipexole	245(33.8)	266(31.9)	0.4147	58(36.3)	453(32.4)	0.3262
Piribedil	196(27.1)	168(20.1)	0.0013	66(41.3)	298(21.3)	<.0001
Entacapone	152(21.0)	109(13.1)	<.0001	43(26.9)	218(15.6)	0.0003
Selegiline	17(2.4)	32(3.8)	0.0931	4(2.5)	45(3.2)	0.6216
Trihexyphenidyl	101(13.9)	114(13.7)	0.8725	17(10.6)	198(14.2)	0.2190
Amantadine	174(24.0)	182(21.8)	0.3000	46(28.8)	310(22.2)	0.0606

*based on patients with complete CRF; *WO* Wearing-off, *UPDRS* Unified Parkinson’s Disease Rating Scale, *LED*, Levodopa equivalent dosage, *ADL* Activities of Daily Living.

### Factors considered when adjusting therapeutic strategies for WO patients

Among 724 patients with WO, 55.7% (*n* = 403) of them needed therapy adjustment according to the opinion of movement disorders specialists. The factors concerned by movement disorder specialists for WO patients, in rank order, were as follows: better improvement of motor symptoms (*n* = 354, 87.8%), long-term disease control and drug selection (*n* = 288, 71.5%), prior consideration of tolerance and safety of medications (*n* = 54, 13.4%), limited economic condition of patients (*n* = 35, 8.7%), the patients’ choices (*n* = 14, 3.5%) and the price of medications (*n* = 11, 2.7%).

### Dyskinesia prevalence and associated factors

Among 1,558 enrolled patients, 160 of them were finally diagnosed with dyskinesia by movement disorders specialists. The overall prevalence rate of dyskinesia was 10.3% (95% CI: 8.8% - 11.8%). Dyskinesia prevalence rate was positively associated with L-dopa treatment duration (*p* = 0.0072) as shown in Figure 2.

Compared with those without dyskinesia, patients with dyskinesia had younger age (*p* = 0.0016), earlier onset age (*p* < 0.0001), lower weight (*p* < 0.0001), longer disease duration (*p* < 0.0001), longer L-dopa therapy duration (*p* < 0.0001), larger amount of L-dopa dosage and LED (*p* < 0.0001) and more disabled ADL (*p* < 0.0001). Subgroup analysis for patients with CRF evaluation showed that up to 88.5% of patients with dyskinesia had WO, which was significantly higher than those without dyskinesia (65.9%) (*p* < 0.0001). In terms of medications, there were more proportions of patients with dyskinesia receiving levodopa-carbidopa (*p* < 0.0001), piribedil (*p* < 0.0001) and entacapone (*p* = 0.0003), in comparison with those without dyskinesia. However, regarding the proportion of levodopa-benserazide, there was no difference between the two groups (*p* = 0.9094) (Table 1).

## Discussion

This cross-sectional, multi-center survey showed that, for Chinese PD outpatients with L-dopa therapy, totally 46.5% and 10.3% suffered from WO and dyskinesia, respectively. Compared with those reported in other populations, the prevalence rate of WO was similar while the prevalence rate of dyskinesia was obviously lower (Table 2). The underlying reasons may be as follows: 1) Methodological differences: in theory, results from current muti-center study and the other three studies [18–20] were more accurate than those from single center reports [2, 4, 5, 11, 21, 22]. The current study revealed WO prevalence (46.5%) in China was a little bit higher than that in USA & Europe (motor fluctuation 43.9%) [19] and lower than that in Czech Republic(66.7%) [18]. 2) Variations on baseline characteristics of enrolled patients: Several prospective studies indicated L-dopa dosage and disease duration were two important predictive factors of the development of dyskinesia [3, 23, 24]. Both items of enrolled patients in the present study were lower than previous reports in North America, Europe and some Asian Countries, which may explain the low prevalence of dyskinesia in China. 3) Race factor and genetic predisposition: Olanow *et al.* found both WO and dyskinesia occurred more frequently in North American versus European sites [24]. It was reported in recent years that several genetic variations (such as COMT Val158Met [25], BDNF val66met [26], mu opioid receptor polymorphism and dopamine D2 receptor intronic dinucleotide repeat polymorphism [27]) were associated with the occurrence of motor complications, especially for dyskinesia. So the specific susceptible loci associated with motor complications in Chinese patients warrant further investigation.

**Table 2:** **Serial epidemiological studies of PD patients with WO and dyskinesia in Asian and Caucasians**

Countries	PD sample	Design	WO prevalence (%)	Dyskinesia prevalence (%)	References
**UK**	87	Community based	40.0(MF)	28	Schrag A, *et al. Brain*, 2000 [2]
**China**	63	Hospital based, single center	44.4	17.5	Liu CF, *et al. Chin J Neurol*, 2003 [5]
**USA & Europe**	289	Hospital based, multi-center	43.9*(MF)	-	Stacy M, *et al. Mov Disord*, 2005 [19]
**Turkey**	555	Hospital based, single center	46.3	30.1	Benbir G, *et al. Clin Neurol Neurosurg*, 2006 [11]
**Chile**	124	Hospital based, single center	52.0	47.2	Juri-Claveria C, et al. *Rev Neurol*, 2007 [21]
**Hong Kong**	98	Hospital based, single center	74.5(MF)	77.6	Kum WF, *et al. J Clin Neurosci*, 2009 [4]
**Czech Republic**	563	Hospital based, multi-center	66.7	-	Bares M, *et al. J Neural Transm*, 2012 [18]
**Japan**	1453	Hospital based, single center	44.7	-	Yoritaka A, *et al. Parkinsonism Relat Disord*, 2013 [22]
**Italy**	617	Hospital based, multi-center	56.9	-	Stocchi F, *et al. Parkinsonism Relat Disord*, 2014 [20]
**China**	1558	Hospital based, multi-center	46.5	10.3	Present study

^*^WO diagnosis according to UPDRS-IV, *MF* Motor fluctuation.

Since WO is generally the first motor complication to develop, early identification of such condition is of great importance for the optimized therapy. This survey showed that approximately 67.5% of Chinese PD patients with more than one positive response of WOQ-9 were finally diagnosed with WO by movement disorders specialists, confirming that WOQ-9 was fit for clinical screening for WO, as recommended by MDS in 2011 [8]. With respect to WO phenomenology, totally, motor WO symptoms outweighed the non-motor WO symptoms, as shown in the present survey and Western Countries’ surveys. Movement slowness was the most common motor WO symptoms, whereas, pain/aching was the most common non-motor WO symptoms in our results. This may be a little bit difference from those in Western Countries. Stacy and co-workers in 2005 found tremor and tiredness were the most frequent motor and non-motor fluctuation symptom, respectively [19]. The potential reason of this discrepancy needs to be further investigated. However, both of these symptoms may provide important information for the identification of WO in clinical practice. It was reported that non-motor fluctuation may involve a greater degree of disability than motor fluctuation [28]. Therefore, more attention should be paid for the recognition of non-motor fluctuation in future research.

Our survey indicated that young onset age, low body weight, large amount of LED, long duration of disease and L-dopa therapy were associated with motor complications in Chinese PD patients with impaired ADL. Other than those with dyskinesia, there were more female and severe motor disabled patients with WO. All these results were consistent with previous cross-sectional investigations by others and a recent prospective study reported by Olanow and co-workers [24]. Based on these studies, clinicians should initiate L-dopa treatment for PD patients with low doses and increase the dosage in small increments in the long run. Besides, it may also be important to consider the weight of patients when prescribing L-dopa. In terms of medications, patients with motor complications took more proportions of L-dopa, piribedil and entacapone. Since this was a cross-sectional study, a proportion of patients may have already employed the adjunctive therapy (such as piribedil or entacapone) for their existed motor complications, we could not draw a conclusion whether these alternative drugs were risk or protective factors of motor complications in the present survey.

As expected, better improvement of motor symptoms (87.8%) and long-term disease control and drug selection (71.5%) were the two frequent considered factors when movement disorders specialists adjusted therapeutic strategies for patients with WO. This may be partially benefited from the clinical practice of Chinese PD treatment guidelines, since published in 2006 and 2009, respectively [9]. The L-dopa treatment concept--using the lowest dose that provides the satisfactory clinical control to delay the occurrence of motor complication, especially among PD patients with young onset age, influenced more and more clinical doctors with its popularity.

There were some limitations of our study. Firstly, patients with no positive response of WOQ-9 did not go to neurologists’ evaluation in the present survey. Besides, only choreatic dyskinesias were counted which could leave out other types of dyskinesia such as dystonic dyskinesias. Therefore, the true prevalence rate of WO and dyskinesia might be a little bit underestimated. Secondly, as a cross-sectional study, only associated factors of motor complications could be found in the present investigation. Since only patients with more than one point of WOQ-9 had UPDRS motor evaluation, we did not perform multiple logistic regression analysis to explore the independent influential factor of WO and dyskinesia. To better clarify its independent predictive factors, prospective cohort studies are needed in future.

## Conclusions

We performed the first multi-center registry survey of WO among Chinese patients with PD on L-dopa therapy. The prevalence rate of WO found in our study was similar to, while dyskinesia was relatively lower than those in other Asian and Caucasian Countries, and different reasons such as methodological difference, L-dopa dosage and genetic susceptibility may contribute to this result.

## Appendix

The following principle investigators participated in the study (Sequence arrangement according to a descending order of included case number): Sheng-Di Chen, Rui Jin Hospital affiliated to Shanghai Jiao Tong University School of Medicine; Ming Shao, The First Affiliated Hospital of Guangzhou Medical College; Tao Feng, Beijing Tiantan Hospital affiliated to Capital Medical University; Wei-Guo Liu, Nanjing Brain Hospital affiliated to Nanjing Medical University; Xiao-Guang Luo, The First Hospital of China Medical University; Xiao-Chun Chen, Union Xiehe Hospital, Fujian Medical University; An-Mu Xie, The Affiliated Hospital of Medical College Qingdao University; Chun-Feng Liu, The Second Affiliated Hospital of Soochow University; Zhen-Guo Liu, Xinhua Hospital affiliated to Shanghai Jiao Tong University School of Medicine; Yi-Ming liu, Qilu Hospital of Shandong University; Jian Wang, Huashan Hospital affiliated to Fudan University; Hui-Fang Shuang, West China Hospital, Sichuan University; Biao Chen, Xuanwu Hospital of Capital Medical University; Bei-Sha Tang, Xiangya Hospital, Central South University; Xing-Yue Hu, Shaoyifu Hospital affiliated to Zhejiang University School of Medicine; Li-Juan Wang, Guangdong General Hospital; Bao-Rong Zhang, The Second Affiliated Hospital of Zhejiang University School of Medicine; Min Ye, Nanjing BenQ medical Center; Hai-Bo Chen, Peking Hospital; Xin-Hua Wan, Peking Union Medical College Hospital; Ming-Wei Wang, The First Hospital of Hebei Medical University; Xian-Wen Chen, The First Affiliated Hospital of Anhui Medical University; Yan Chen, General Hospital Affiliated to Tianjing medical University; Guo-Guang Peng, The First Affiliated Hospital of Chongqing Medical University; Zhen-Fu Wang, The General Hospital of Chinese People’s Liberation Army; Ping-Yi Xu, The First Affiliated Hospital, Sun Yat-Sen University; Sheng-Gang Sun, Union Hospital of Tongji Medical College of Huazhong University of Science and Technology; Xiang-Ru Sun, The First Hospital affiliated to Beijing University.

## Electronic supplementary material

Additional file 1: Table S1: Demographic and basic clinical information of enrolled patients and those with complete CRF. (DOC 50 KB)

## Abbreviations

PD: Parkinson’s disease
WO: Wearing off
WOQ-9: 9-item wearing off questionnaire
COMT: Catechol-O-methyltransferase
MAO-B: Monoamineoxidase-B.
