# Supplementary material for: Prevalence of wearing-off and dyskinesia among the patients with Parkinson’s disease on levodopa therapy: a multi-center registry survey in mainland China
Source: Transl Neurodegener. 2014 Dec 5;3:26. doi: 10.1186/2047-9158-3-26 (PMC4323338; doi:10.1186/2047-9158-3-26)
Supplement: Supplementary file 1 — Additional file 1: Table S1: Demographic and basic clinical information of enrolled patients and those with complete CRF. (DOC 50 KB) [file 40035_2014_73_MOESM1_ESM.doc]

**Supplementary table 1. Demographic and clinical information of enrolled patients and those with complete CRF**

| **Items** | **Enrolled patients**  **（*n* = 1558）** | **Patients with complete CRF**  **（*n* = 1051）** |
| --- | --- | --- |
| **Age, years**  Mean ± SD  *n* (missing) | 65.2±9.5  1525(33) | 64.1±9.3  1024(27) |
| **Gender, Female**  *n* (%)  *n* (missing) | 703(45.2)  1556(2) | 495(47.1)  1051(0) |
| **Height, cm**  Mean ± SD  *n* (missing) | 164.4±7.8  1543(15) | 164.2±7.8  1040(11) |
| **Weight, kg**  Mean ± SD  *n* (missing) | 61.6±10.9  1546(12) | 61.4±11.0  1044(7) |
| **Onset age, years**  Mean ± SD  *n* (missing) | 59.9±10.0  1494(64) | 58.3±9.9  1025(26) |
| **Disease duration, years**  Mean ± SD  n (missing) | 5.4±4.3  1509(49) | 6.0±4.3  1038(13) |
| **L-dopa duration, years**  Mean ± SD  *n* (missing) | 4.1±3.8  1488(70) | 4.5±3.8  1008(43) |
| **UPDRS-III**  Mean ± SD  *n* (missing) | - | 17.92±8.1  1051(0) |
| **WOQ-9 score**  Mean ± SD  *n* (missing) | 2.4±2.2  1558(0) | 3.6±1.8  1051(0) |
| **mAIMS score**  Mean ± SD  *n* (missing) | - | 1.9±4.7  1005(46) |
| **ADL**  Mean ± SD  *n* (missing) | - | 26.1±12.5  1047(4) |
| **L-dopa dosage**  Mean±SD  *n* (missing) | 413.1±227.8  1558(0) | 444.0±246.3  1051(0) |
| **LED**  Mean±SD  *n* (missing) | 481.3±258.8  1558(0) | 520.2±278.7  1051(0) |
| **Medications for PD, *n* (%)** |  |  |
| Levodopa-benserazide | 1329(85.3) | 916(87.2) |
| Levodopa-carbidopa | 538(34.5) | 395(37.6) |
| Pramipexole | 511(32.8) | 370(35.2) |
| Piribedil | 364(23.4) | 264(25.1) |
| Entacapone | 261(16.7) | 210(20.0) |
| Trihexyphenidyl | 215(13.8) | 154(14.6) |
| Amantadine | 356(22.8) | 237(22.6) |
| Selegiline | 49(3.2) | 26(2.5) |

*UPDRS* Unified Parkinson’s Disease Rating Scale, *WOQ-9* 9-item wearing-off questionnaire, *mAIMS* Modified Abnormal Involuntary Movement Scale, *LED* levodopa equivalent dosage, *ADL* Activities of Daily Living.
